# Social and ecological challenges of market-oriented shrimp farming in Vietnam

**DOI:** 10.1186/2193-1801-2-675

**Published:** 2013-12-17

**Authors:** Ngo Thi Phuong Lan

**Affiliations:** University of Social Sciences and Humanities, Vietnam National University, Ho Chi Minh City, Vietnam

**Keywords:** Shrimp farming, the Mekong Delta of Vietnam, Market-oriented production, Risky business, Social and ecological challenges, Labor migration, Risk mitigation

## Abstract

Vietnam is one of the largest shrimp exporters in the world. Since 2010, Vietnam has earned about two billion dollars annually through shrimp exports. As a fertile area of greatest potential for agricultural production in Vietnam, the Mekong Delta has been a major contributor to the country’s achievements, especially in the agricultural sector. During recent decades, trade liberation along with various policies in support of aquaculture has accelerated the development of shrimp production in the Delta. Based on an ethnographic study of shrimp farming in the Mekong Delta of Vietnam, I assert that along with great rewards arising from the expansion of shrimp farming areas, productivity, and export value, the shrimp industry has brought various environmental, economic and social challenges. Consequently, shrimp farming is a risky business and local inhabitants have relied on various strategies to cope with these challenges. Risk mitigation in shrimp production and labor migration are the two important strategies of local inhabitants for securing their livelihoods. Water pollution and poor quality post-larvae shrimp are direct consequences of market-oriented production.

## Background

Shrimp production has emerged as one important livelihood in the Mekong Delta of Vietnam. It promises to bring great rewards to farmers who have shifted from rice cultivation to shrimp farming. In the context of a sharp increase in shrimp farming area and the number of households engaging in this industry, I carry out this research to examine how this kind of livelihood affects the region and in the context of present practices of shrimp farming whether this is a sustainable livelihood for farmers.

## Introduction

As a fertile delta having the greatest potential for agricultural production in Vietnam, in the context of increasing integration into the global market, the Mekong Delta has contributed greatly to the achievement of Vietnam’s agricultural outputs. Under the impacts of various policies, trade liberation, and technological improvement, agricultural activities in the Delta are increasingly market-oriented. Farmers in the Delta have increasingly cultivated cash crops and become engaged in the global market. High-valued cash crops have increased in scale and intensity in a region where land is under de-facto private ownership. Consequently, although the Delta constitutes only 12% of the country’s area; 30% of national agricultural land; and 21% of the total population, annually, the Mekong Delta contributes 50% of rice production; 90% of exported rice; 80% of aquaculture production; 60% of exported aquaculture products; 60% of total exported products for the country; and it contributes 18% towards the nation’s GDP (Guidance Board of Vietnam’s Southwest region, Ban Chỉ đạo Tây Nam Bộ và Trung tâm thông tin Sài Gòn 2005, p.17). Along with this economic growth at macro level, the Mekong Delta has undergone significant changes at the micro level, particularly with respect to livelihoods of the Delta’s inhabitants.

During recent decades, structural transformation in agriculture has become a prominent phenomenon in the Mekong Delta. Technological advances which provide high-productivity plant and animal breeds, new fertilizers and pesticides have played a crucial role in the transformation of the region. Further structural transformation in the Mekong Delta’s agriculture has taken place with the shift from traditional rice cultivation to new agricultural products like fruit and aquaculture. In particular, the shift to export-oriented shrimp farming has been accelerated. For example, in Ca Mau province in the lower Mekong Delta, the shrimp cultivation area increased from 90,551 ha in 1999 to 239,398 ha in 2003 and 257,000 ha in 2008 (Mai Trong Thong et al. 2006, p.7; information from Ca Mau province People’s Committee). The rapid development of the shrimp industry in the Mekong Delta has had considerable influence on the Delta’s economy, society and ecology.

### Shrimp farming in Vietnam: market-oriented production

With 3,260 km of coastline, Vietnam is a suitable place for brackish aquaculture (Le Ba Thao 2002, p.8). According to statistics from the Ministry of Agriculture and Rural Development (MARD) of Vietnam, Vietnam had 613,000 ha under shrimp production in 2010. Because of a wide discrepancy in temperature between seasons and low temperatures in winter, the northern part of Vietnam is not suitable for raising shrimp all year round. According to the Fisheries Informatic Center of the Vietnamese Directorate of Fisheries, sea and brackish water shrimp in the northern parts of Vietnam are raised in Hai Phong, Nam Dinh, Thai Binh, Ninh Binh, and Quang Ninh provinces. The central part of Vietnam is known both for shrimp farming and hatcheries. In this region, shrimp farms are concentrated in the provinces of Thanh Hoa, Nghe An, Ha Tinh, Quang Binh, Quang Tri, Ninh Thuan, Binh Thuan, Da Nang, Quang Nam, Quang Ngai, Binh Dinh, Phu Yen and Khanh Hoa. Khanh Hoa province is considered as a cradle of the shrimp farming movement thanks to its successful experiment in artificially producing shrimp fry in the 1980s. Presently, Khanh Hoa is still a well-known centre for shrimp hatcheries in the country.

In the south of Vietnam, shrimps are raised in the southeast region (Ho Chi Minh City, Dong Nai, Ba Ria – Vung Tau provinces), and in the Mekong Delta (Long An, Tien Giang, Ben Tre, Kien Giang, Tra Vinh, Soc Trang, Bac Lieu, Ca Mau provinces). Given its appropriate soil and climate conditions, the Mekong Delta, which is naturally bestowed with a dense network of rivers and ditches, fertile alluvial plains, and diversified mangrove forest, produces 75% of the country’s shrimp products (GSO 2009, p.344). According to the Ministry of Agriculture and Rural Development, in 2008, with 539,607 ha, the Mekong Delta was the biggest shrimp producing region in Vietnam and accounted for approximately 89.3% of the country’s shrimp area. Among eight provinces, Ca Mau has the largest area (50% of the region’s total area) dedicated to shrimp raising. Results from the *2006 Rural, Agricultural and Fishery Census,* conducted by the Vietnam General Office of Statistics, also confirm the importance of the Mekong Delta in terms of the quantity of shrimp farming households. Out of 337,614 households in Vietnam engaged in shrimp farming, 292,522 (86%) of households were from the Mekong Delta. The average size of shrimp farming land of 53% of these households was 0.5 ha, and only 16% of these households engaged in intensive and semi-intensive shrimp culture while 87% of shrimp farming households in the central part of Vietnam engaged in this type of shrimp culture (GSO 2007, p.436, p.437, p.448, p.449, p.453).

The years 2000 to 2002 were a landmark period for the shrimp industry in Vietnam as well as in the Mekong Delta with a dramatic increase in shrimp farming areas and production. Vietnam produces both fresh-water shrimp and brackish-water shrimp but the latter accounts for 97% of the total production. Vietnam’s shrimp production increased from under 200 tons in 1976 to 158,755 tons per annum in 2001 (cited in EJF 2003, p.4). With this boom, since then Vietnam always entered the top five leading shrimp exporters in the world. This achievement is a result of various national policies, starting with the Doi Moi policy and various subsequent policies in support of shrimp farming. Current major export markets for shrimp from Vietnam are the United States and Japan.

Shrimp raising in the Mekong Delta is widely said to have emerged in Ca Mau province in the 1980s when farmers spontaneously let saline water into rice fields to raise shrimp naturally. However, some reports indicate that rice-shrimp rotation farming was applied in the 1960s (Vuong Do Quang and Lin 2001, p.4) and Soc Trang province was believed to be the first place to officially adopt this model in late 1980s (interviews with leaders of Soc Trang province). Although being officially permitted to raise shrimp in their rice fields by Resolution No. 09 in 2000 and Decision 173/2001/Qđ-TTg in 2001, shrimp farming in specific parts of the Mekong Delta provinces was given official permission at different times. For example, in Ca Mau province, shrimp were officially raised large-scale in mangrove forests as a commodity in 1992 (Vo Thanh Binh 2006, p.13); in Bạc Lieu province in 1998 (Guidance Board of Vietnam’s Southwest Region Ban Chỉ đạo Tây Nam Bộ và Trung tâm thông tin Sài Gòn 2005, p.119), and in Long An province in 1994. Moreover, as a result of the locally specific implementation of national policies, each locality has its own trajectory of transformation from rice to shrimp cultivation. In Long An (the case of Tan Chanh commune), for example, the shift was gradual, while in Ca Mau (the case of Hoa My commune) it happened suddenly and simultaneously. Thanks to high value of shrimp, with favorable conditions, the Delta’s farmers have rapidly shifted to this new livelihood. For example, when farmers were officially allowed to raise shrimp in rice fields, the shrimp-raising acreage of Cai Nuoc district, Ca Mau province increased by 8.5 times, reaching 65,000 ha in the period from 1997 to 2002 (Mai Trong Thong et al. 2006, p.8). In Can Duoc district of Long An province the shrimp-raising acreage increased by 5.6 times with 935 ha in the period from 1995–2002 (Tan Chanh’s people committee, 2009).

In the Mekong Delta, the majority of farmers apply extensive and semi-extensive types of shrimp culture. According to the Ministry of Agriculture and Rural Development, in 2008, only 8% of the Delta’s shrimp area was used for intensive shrimp culture while the rest of shrimp area consisted of extensive and advanced-extensive methods of shrimp raising. Research undertaken in 2009 and early 2010 in Hoa My commune of Ca Mau province showed 91% of the shrimp-farming households (n = 202) studied adopted extensive shrimp culture, the remaining 9% of households engaged in intensive and semi-intensive types of culture. In Tan Chanh, the biggest shrimp producing commune in Can Duoc district of Long An province, advanced extensive or semi-extensive culture was adopted by 100% of the households (n = 152) studied.

In principle, the density of shrimp post larvae is from three to five shrimps per m^2^ under the advanced-extensive method of shrimp production. However, in Long An province, in order to have high productivity despite limited land, people release shrimp fry into their ponds with a much higher stocking density (from 10 to 20 fry per m^2^) than the level recommended for extensive shrimp farming. Besides the two aforementioned types of shrimp culture, a minority of famers in both study sites also practice industrial shrimp farming which requires a much higher capital investment per ha per year, normally 40 times higher than the extensive type of farming and five times higher than the advanced-extensive type (calculated from research in the Ca Mau province study site). In contrast to other types of shrimp culture in which shrimp fry are released and harvested once per crop, in the extensive type, people usually release fry once every one to two months and harvest shrimp every day or every one to two weeks.

Presently, people mainly raise black-tiger shrimp (*Penaeus Monodon*), although white-legged shrimp (*Penaeus Vannamei*) have also been raised recently. The latter type of shrimp was introduced in Central Vietnam in 2000 and permitted to be raised in the Mekong Delta only in 2008. Although they grow more quickly and have a higher yield, white-legged shrimp usually cause extreme pollution to shrimp ponds due to their higher waste excretion. Thanks to their high effectiveness in terms of high yield, although being previously claimed to be a potential cause of disease outbreak and harmful to other species, white-legged shrimp are under consideration for removal from the list of harmful species and considered as an appropriate species for commercial production.

Along with this dramatic shifting in livelihoods, coastal areas in the Mekong Delta are witnessing emerging socio-economic and environmental challenges, for which people have to adopt various strategies to cope. Given these challenges, the promising shrimp industry becomes a risky business.

### Ecological and socio-economic challenges amongst shrimp farming communities in the Mekong Delta

The booming shrimp industry, which relies on saline water ecology, has been associated with serious environmental and social impacts. Some studies report that globally environmental problems associated with shrimp farming include destruction of mangroves and other wetland habitats; dispersion of chemicals and nutrients into the environment; pollution and salinity; and the depletion and biological contamination of wild fish and shrimp populations. Social impacts associated with shrimp farming include the increase of poverty and landlessness, food insecurity, and impacts on health and education (EJF 2003, 2004; Barraclough and Finger-Stich, 1996; Preston and Clayton 2003). In this part, drawing primarily on information from current research amongst the two aforementioned research study sites of Hoa My commune and Tan Chanh commune, the dynamics of these ecological and socio-economic challenges of shrimp farming in the Mekong Delta will be examine in detail.

#### Ecological challenges

With the shift from rice to shrimp farming, local ecologies and landscapes of rural communities have experienced tremendous changes. In the two brackish-water study site communities that have now adopted the practice of rice-shrimp farming, inhabitants’ past living patterns revolved around rice cultivation during the rainy season. Their leisure time was in the dry season. At present, the rhythm of farmers’ lives is different. They mainly raise shrimp in the dry season and their leisure time is in the rainy season. In order to raise shrimp, people have converted their rice fields, residential land, or gardens into shrimp ponds. Commune landscapes are no longer characterized by green or yellow rice fields but treeless and spacious brackish shrimp ponds. The system of dikes and dams preventing saline movement was removed, while many canals were enlarged or newly dug. As a result, a dense and vast system of canals characterizes shrimp-farming communities, especially those engaging in extensive shrimp culture. Various fruit gardens no longer exist partly due to their clearance for shrimp ponds and partly due to saline intrusion. Nipa palms used to be grown across large areas of brackish-water communities to supply roofing materials for houses. Now, this kind of plant occupies only a small portion of the landscape, partly because of the space given over to shrimp ponds.

The diversity of local wild aquatic species is also affected by shrimp farming. At the two study sites of Hoa My and Tan Chanh communes, the practice of using poisonous chemicals to kill various fish competing with shrimp for food and killing shrimp fries in shrimp ponds contributes seriously to the decrease in wild species in these areas. Farmers report that many species of local fish have decreased dramatically, in type and quantity, owing to this practice.

Soil and water pollution is another serious problem associated with the shrimp industry. Besides the poor quality of shrimp fry, pollution is said to be an important cause of continuous shrimp losses. The practice of releasing high stocking densities of shrimp fry into ponds to maximize profits under the practice of intensive and semi-intensive types of shrimp culture, often leads to high levels of feeds, chemicals, and antibiotics being used in ponds. Effluence including chemical inputs and waste from shrimp farming ponds are often released directly into the natural environment without any treatment, even in the case of shrimp disease outbreaks. This is a direct source of contamination to soil, rivers and coastal habitats. Moreover, theoretically, it is good to raise shrimps in the dry season. However, because of repeated shrimp losses, farmers want to make up for their lost shrimp crops quickly and often expand the shrimp-raising time beyond the dry season. As a result, farmers raise shrimp throughout the year by keeping saline water in their ponds even in the rainy season. Unlike scientists who can figure out the scale of pollution by scientific measures, farmers measure this problem by observing their shrimp products. Farmers reported that in the past when only a few people engaged in shrimp culture and shrimp were raised using low stocking densities, the shrimp-raising environment was good. Even though they did not pay much attention to taking care of shrimp or apply many technological measures, farmers could succeed remarkably with low investment. Now, with many farmers raising shrimp more intensively by increasing stocking density and the frequency of shrimp crops, despite being backed by advanced technology and aquatic engineers, farmers still frequently lose their crops. Moreover, the practice of using prohibited harmful chemicals that rapidly and effectively kill competing species or stimulate shrimp to shed their exoskeleton so that they grow more quickly, is said to cause certain damage to the ecosystem of shrimp farming areas. However, because of their low expense and lack of immediate impact to shrimp ponds, this practice of chemical use is still widely adopted by shrimp farmers in the Mekong Delta.

Farmers often blame their shrimp losses on the pollution caused by the intensification of shrimp farming. However, while they have limited land and suffer continuous shrimp losses, they still continue the practice of intensification because of their wish to increase the productivity. As a result, in order to raise shrimp intensively, farmers increasingly rely on technology in terms of food and chemicals that are direct sources of pollution to the shrimp environment. Shrimp disease outbreak has become a prominent and growing concern of shrimp farmers. Reports show that in the Mekong Delta failure rates in shrimp farming as high as from 70% to 80% started in 2001 (EJF 2003, p.17) when Vietnam began to promote large-scale shrimp production. The case of Tan Chanh commune in Long An province best illustrates this point. Although shrimp farming was adopted in 1994–1995, it was not until the period of 2000–2001 that this practice was widely adopted by the locals. The period of 2000–2001 was also considered a “golden age” for the shrimp industry with remarkable rewards to local shrimp farmers. Thanks to big profits from shrimp, the infrastructure of the community was being upgraded. Many concrete and durable houses were built in the community because of shrimp farming. However, along with the increase of shrimp farming land and intensive production, shrimp disease outbreak has since become more and more prevalent in the community. It affected 60% of shrimp farms in 2008. This number increased to 80-90% in 2009 and 2010 although farmers were reported to strictly observe instructions relating to the crop calendar, pond treatment and seed selecting. This was a huge loss because annually the shrimp industry brings about 20 billion VND (1 million USD) to farmers in Tan Chanh commune in a good harvest year.

Another ecological challenge to shrimp farmers is the effect of salinity. Attracted by extremely high profits of shrimp in comparison to rice, a majority of farmers engage entirely in shrimp farming instead of practicing rice-shrimp rotation which was encouraged by local governments as a friendly and sustainable livelihood. In Long An province, people have tried their utmost to use land for shrimp ponds. They have made use of every single acre of residential land to make shrimp ponds. Consequently, shrimp ponds are located very close to farmers’ houses. At first, residents still cultivated rice to secure their food supply while raising shrimp. However, with the striking success of the first shrimp crops, farmers stopped cultivating rice to save time and energy for shrimp raising. Shrimp are raised year round, while rice fields are abandoned because rice is no longer considered as important as shrimp. As many farmers have not continued to grow rice, it has become difficult for other farmers to continue growing rice because of the invasion of rice crop destroyers such as insects, birds, and rodents. Gradually, having limited land and wishing to increase shrimp production, a majority of farmers removed the rice fields in their land to make the ponds deeper so that they could raise more shrimp. This means that it is now impossible for these farmers to grow rice anymore. Overall in the Long An province study site, after suffering serious repeated shrimp losses, farmers cannot return to rice cultivation for their food security. In Ca Mau province, because of much land is dedicated to extensive shrimp raising and the high expense of turning rice fields into ponds, farmers tended to keep saline water for longer periods in their ponds to raise shrimp in an effort to regain some of the outlay costs encountered from the initial conversion of their rice cultivation land into shrimp ponds. With the increase in salinity caused by the practice of keeping saline water in shrimp ponds, rice yields were not high and plants were even destroyed by saline water. Consequently, in Ca Mau province, rice farmers have to invest a great amount of labor and energy to keep their rice from crop destroyers and face a standing danger of salinity intrusion from canals or neighboring shrimp ponds where now the priority is keeping saline water in the fields.

The environmental challenges of shrimp production can also be seen from the aspect of the quality of shrimp. In the shrimp industry, intensive production leads, not only, to a polluted environment but also to the low quality of shrimp fry. The quality of post larvae stock plays an important role in the success of shrimp crops and farmers are unable to control this determinant. There is an upsurge in demand for shrimp fry with the boom in the shrimp industry while the infrastructure of shrimp hatcheries has not developed adequately. High demand and limited supplies have resulted in poor quality control systems. Broodstock ideally breed only once or twice and from parent shrimp taken from the open sea. However, in reality, to meet the high demand, parent shrimps are forced to overreproduce from six to eight broods. Later broods tend to be of poor quality with high mortality rates and increased susceptibility to disease. Due to limited natural parent shrimp, raised parent shrimp are chosen to produce broods which are also of poor quality.

In short, market-oriented production in the shrimp industry leads to serious ecological tensions which, in turn, negatively affect the industry itself and the livelihoods of inhabitants.

### Socio-economic challenges of shrimp farming: problems of unequal access and household production

#### The problem of unequal access to shrimp farming

As a risky business, shrimp industry development is associated with newly emerging socio-economic tensions among shrimp farming communities in coastal areas of the Mekong Delta. With high returns, the shrimp industry has become an attractive business for many people who are capable of high capital investment. Therefore, although shrimp farming is the primary new livelihood practice amongst coastal communities of the Mekong Delta, studies show that this industry has not been viewed as an equal access industry for all local people (EJF 2003; Le Thi Van Hue and Scott 2008; Pham Van Khang 2008). Especially, in communities that adopt intensive shrimp culture, local people, mainly the poor, are excluded from this industry due to its requirement for high capital investment. Instead many local poor people have sold or leased their ponds instead of participating in shrimp farming themselves. Consequently, shrimp ponds are owned by outsiders, usually governmental officials or the rich (EJF 2003). In Soc Trang and Ben Tre provinces of the Mekong Delta, many local famers sell land to wealthier people who come from other places to invest in intensive shrimp culture (Pham Van Khang, 2008and researcher’s own observations in Soc Trang province). It is reported that this phenomenon is also common in Ninh Thuan province of Central Vietnam, where only 96 out of 250 shrimp farmers are locals (Soo Kum Lin 2006, p.2).

Ironically, unequal access is not always bad for the locals. Reports show that due to the high risks of shrimp farming, the poor have became least indebted and relatively economically unaffected because of their lack participation. Meanwhile the rich, the upper middle and middle income households who are engaged in shrimp farming have lost their investment, plunging them into serious debt (Le Thi Van Hue and Scott 2008; Luttrell 2006). In Long An and Ca Mau provinces, despite knowing that intensive shrimp farming can bring great rewards, a majority of farmers prefer practicing extensive and semi-extensive types of shrimp culture rather than the intensive type because of its extremely high risk.

With the participation of outsiders who have enough capital to invest in the risky business, consequent landlessness is often considered as a negative result of the shrimp industry (Luttrell 2006; EJF p.26; EJF 2003; Le Thi Van Hue and Scott p.17; Le Thi Van Hue and Scott 2008, p.70, Pham Van Khang 2008, p.45-47). However, outsider participation in shrimp farming *is not always an issue*. In the two research in Long An and Ca Mau provinces, where extensive and advanced-extensive types of shrimp culture are widely adopted, shrimp farmers are mostly local people who had previously cultivated rice. During the golden age of shrimp farming, land values in these localities increased. Outsiders who had capital and knowledge of raising shrimp (mainly aquaculture engineers) came to these sites to invest in shrimp farming. However, it was not easy for them to purchase land in these communities because most local landowners were reluctant to give up their land entirely due to their historical and cultural ties to the land. Therefore, those coming from elsewhere to invest in shrimp aquaculture had to rent ponds to raise shrimp. Often after several unprofitable crops, they then had to quit. In contrast to other studies, which claim landlessness as a negative impact of shrimp farming (EJF 2003; Luttrell 2006; Le Thi Van Hue and Scott, 2008), this study shows that landlessness due to shrimp farming *has not been a widely common phenomenon* in shrimp farming communities, especially in those places where farmers adopt extensive and semi-extensive types of shrimp culture. To farmers, land is an economically and culturally valuable asset. Selling off their land is the last resort for farmers. During hard times, they resort to many strategies to keep their land. For example, at the beginning stage of shrimp farming, when the poor could not engage in shrimp farming, they either, rented their land out to others and took their land back when they could afford shrimp farming, or left their fields vacant while engaging in off-farm jobs to earn their living. In shrimp production, although incurring bad debt is a pervasive phenomenon, selling land, the only valuable asset of most farmers, to pay off their debt is not always the case. In the case that they cannot pay back loans or debts resulting from shrimp farming, they normally pledge their land (*cấm cố*) to their local neighbors who will give them some money (in gold equivalence) to use that plot of land until the land owners are able to pay them back that same amount of money (in gold equivalence with no interest charged). Failing shrimp farmers either migrate to other places to find jobs or rent back that same piece of land to raise shrimp in order to accumulate money to be able to get their land back. My study also shows that although loans from government banks with favorable interest are the most important source of capital that help people to take part in shrimp farming, other informal types of support from relatives, neighbors (in the form of cash, labor, and land) and people within the shrimp market network (buying food or shrimp post-larvae on credit) are also important, especially to the poor.

 (Akerlof In shrimp farming, my study shows that the opportunity for access to high quality post-larvae stock is unequal. The poor quality control system and the nature of small-scale household production prevent a majority of shrimp farmers from accessing good quality shrimp fry. Only wealthy farmers who often adopt the intensive type of shrimp culture can afford the expenses of shrimp fry quality control. Other farmers, who often buy a small quantity of shrimp fry, are unable to demand high quality post-larvae stock. It is the fact that poor or small-scale farmers often buy post larvae stock that cannot be sold to wealthy farmers. Farmers have to rely on their network of brokers for their shrimp fry. In this type of market set-up, they have no power to negotiate and examine the quality of post-larvae shrimp and can actually be considered a *lemon market*^1970^).

Unequal access to shrimp production can be analyzed not only from the aspect of economic stratification but also from the standpoint of social class and ethnicity. In shrimp raising communities, because shrimp farming is a new practice that requires complicated technology, local governments have tried to help farmers by providing various forms of training. Training courses and broadcasting programs are the two most common channels of disseminating knowledge of raising shrimp to farmers. Due to the limitation of government resources to carry out these tasks, members of various unions (the farmers associations, the women associations and the veteran associations) are mainly selected to participate in these training courses. Moreover, in the Mekong Delta, the Kinh are not the only ethnic group engaging in shrimp farming. In the Delta, Khmer people make up a significant portion of the population. They are not good at Vietnamese language skills; language becomes a barrier for the Khmer in the Mekong Delta for accessing shrimp raising knowledge which is often provided in the Vietnamese language. Khmer shrimp-raising farmers in Cau Ngang district of Tra Vinh province reported that they did not receive any help from the local government in raising shrimp while, in reality, there were various shrimp-aid programs provided by the local government. Without access to shrimp-raising knowledge, many Khmer farmers in Cau Ngang district suffer serious loss which pushes them to quit shrimp farming, leaving their shrimp ponds vacant and migrating to other places to earn their living and to pay off their debt resulting from shrimp farming.

In the shift from rice cultivation to shrimp farming, unequal access to shrimp farming can be analyzed through the fact that farmers are not always free decision makers. In the decision-making process to raise shrimp, farmers play both an active and passive role. The phenomenon that all farmers in coastal areas are shrimp producers may imply that shrimp farming is an equal access opportunity to all local people. However, this study shows that although the potential to earn a high profit is a decisive factor to urge a majority of people to shift actively into shrimp farming, farmers are not always free-decision makers in this process. Some households were reluctant to shift to shrimp farming because they did not want to take on the risks involved with shrimp farming. Some of them were already living at the subsistence margin. Those reluctant to take on shrimp farming engaged in shrimp farming because they did not have any other alternative because, with the shift to shrimp farming, the irrigation system has favored saline water. They could not grow rice, the traditional crop from which they already had a stable income, while their neighbors were raising shrimp. Saline water intrusion from neighboring shrimp ponds was a standing danger for their rice crops. Results from the survey on structural transformation of agriculture-forestry-fisheries in the 1999–2005 period conducted by the Ca Mau People’s Committee confirm this phenomenon. This report shows that in Cai Nuoc district of Ca Mau province, among the reasons for converting from rice to shrimp, aside from the reason to improve households’ economic conditions, residing on planned areas for shrimp farming was also an important reason, accounting for 43% of the surveyed households (Ca Mau’s People Committee 2005, p.49). Having other sources of income and the possibility of mobilizing social capital embedded in various social organizations in terms of financial capital, labor, and land helps poor farmers to participate in shrimp farming.

#### Household production in shrimp farming: a challenge to sustainable production

Households are both a social and economic unit where people are linked by kinship and marriage while performing economic activities together. Due to these overlapping characteristics, it is very difficult to represent the relationship between the household economy and market economy because these two concepts are often put as opposed to one another. The implication of this opposition is that because the household produces by itself what it needs, it does not need the market (Enrique 2005, p.405). In the case of shrimp farming in Vietnam, most shrimp farms are small-scale, relying on the labor source from members in the family. With the shift to shrimp farming, households are no longer associated with a subsistence economy but market-oriented production. In brackish areas of the Mekong Delta, before farming shrimp, farmers produced rice. However, due to unfavorable soil conditions, rice crop yields were not high. Therefore, a majority of farmers in these areas could not participate actively in market production. In Long An, for example, rice production was mainly for the household’s subsistence. In the former rice-cultivation period, local inhabitants had practiced a subsistence economy in which they farmed rice, vegetables, fruits, pigs, poultry, and fish for their own consumption for the entire year. Thanks to the fresh-water ecology, people could also catch various wild fresh-water aquatic varieties, which were abundant in canals at that time. According to local government reports in Tan Chanh commune of Long An province, during the rice-cultivation period, 94% of households were involved in rice growing, 4% were involved in wild capture fisheries, and 30% were involved in fish-raising. Poultry, pigs and buffaloes were also raised to sell in the locality. Farmers could sell the excess volume of these products for cash to cover their families’ other expenditures such as ceremonies, health care, and education. In the shrimp-production period, all household needs, including rice as the staple food, are met mainly through earnings made from market-oriented shrimp production. In coastal areas of the Mekong Delta, in the rice-cultivating era, rice was mainly produced for local inhabitants’ consumption. In contrast to rice, at present, shrimp are mainly produced for the market. Shrimp farming has become the main agricultural activity with 80% of the studied households in Long An Province and 94% in Ca Mau Province involved in shrimp-raising. Due to the high risk of shrimp farming which cannot ensure a stable source of income, local inhabitants have experienced high levels of food insecurity. Without rice, other activities associated with rice such as animal husbandry also no longer exist. Going to markets (in Long An province) or buying from trading boats (in Ca Mau Province) for their meals have become a daily activity of shrimp farmers. Debt owed from buying food are still prominent in shrimp farming communities with local grocery stores still playing an important role in “helping” local inhabitants for their food security by selling food on credit and waiting for the harvest time of shrimp before receiving payment from customers. “Eating in advance, paying back later” is a pervasive motto in shrimp farmers’ lives.

De facto private ownership of agricultural land proves to be a favorable factor that helps shrimp farmers to participate in market-oriented production. Although land remains the property of the state, usage rights can be legally transferred and exchanged, mortgaged and inherited. Farmers have the right to decide how to use their land for production. In shrimp farming, with the government’s encouragement, farmers have eagerly converted their rice fields into shrimp ponds with the hope of improve their standard of living. Although private ownership of land is an advantageous condition in agricultural production, due to small-scale household production, shrimp farmers are finding sustainable production a difficult challenge to meet.

Having limited land is a tension that motivates shrimp farmers to raise shrimp more intensively. Farmers try to stock at higher densities and increase the frequency of their shrimp crops. Like other agricultural activities, shrimp farms are operated by households whose cultivated lands vary in size. Although it is reported that a majority of shrimp–farming households own cultivation land ranging from 0.5 to 2 hectares in size (50.58% for the whole country and 53.48% for the Mekong Delta) (GSO 2007, p.448-449), this survey in two provinces, one with the largest shrimp cultivation areas per household and the other with the smallest shrimp cultivation areas per household, shows a high variability in the size of shrimp farms. In Ca Mau province 55.3% of the households surveyed in Ca Mau province own over 1 hectare of shrimp farming land while the figure is only 6.9% in Long An provinces. 5 hectares and 0.068 hectares are the largest and the smallest shrimp areas of households studied in Ca Mau province while the equivalent measures are 1.3 hectares and 0.03 hectares in Long An province respectively.

However, farm sizes may not inform the scale of capital investment because it depends on the type of culture. In my study, people mainly adopt extensive and semi-extensive shrimp farming in which shrimp acreage is a good indicator for the source of income. In Long An province, the average size farm is 0.4 ha while in Ca Mau the average size farm is 1 hectare. According to Vietnamese standards, these farm sizes are classified as a lack of cultivated land (Nguyen Dinh Huong 1999). It means that the income generated from these pieces of agricultural land cannot guarantee the household’s subsistence. Income from a shrimp pond of a medium sized family with five people illustrates more details:

“My family has raised shrimps since 1996. My cultivation land is 5,000 m^2^. In the first year, we earned nine million dong (equivalent to 750 USD), the next year 47 million dong (3,900 USD). This was the best crop that helped us to rebuild our house. From 2000 to 2003, we got on average 13 million dong (870 USD) annually. From 2004 to 2007, we suffered repeated losses or earned nothing. We lost from five to seven million dong (310–440 USD) each year. In 2008 and 2009, we earned 10 million dong (540 USD) annually.” N.T.T.M, female, aged 35, Long An province, shrimp farmer

Profits from shrimp are much higher than from rice, normally from five to seven times with the same cultivated land. By raising shrimp, farmers can obtain large incomes that they “have never dreamed of when cultivating rice”. However, due to the high risks in connection with price fluctuations, a changing environment, and disease outbreaks, this kind of production is no longer considered the main source of income for local inhabitants. A majority of farmers raise shrimp as one way to try their luck. In shrimp farming, it is very easy to make a loss or earn nothing. The case study of the above-mentioned household with an average-sized shrimp pond in Long An province shows that income generated from shrimp is not enough for a household with five people who at present have to rely mostly on the market for their daily consumption because their subsistence economy no longer exists. Farmers have various strategies to survive. In shrimp production, those engaged in advanced extensive types of shrimp culture have increased the shrimps’ stocking density and raise shrimp throughout the year, while those engaged in extensive type of shrimp culture have raised various kinds of fish in shrimp ponds and try hard to adopt rice-shrimp rotation to ensure their food supply. When farmers cannot adapt locally, labor migration is their best solution to maintain their living. Labor migration has become a prominent phenomenon in shrimp farming communities especially after repeated shrimp losses. Both men and women migrate to industrial centers or other areas to find jobs. In Ca Mau province, while young people tend to travel far distances to Ho Chi Minh city, Dong Nai and Binh Duong provinces of the southeast region of Vietnam, other people tend to work locally with various manual jobs. In Long An province, a large wave of young rural women going to work in garment factories is partly a result of unsustainable production of shrimp farming.

Although local governments with various support policies have an important role in promoting shrimp cultivation, spontaneity is a common feature in shrimp production. There are no official or non-official organizations relating to shrimp farming at the community level. Households do their shrimp farming independently. As a result, individualist rationality is seemingly good at the household level but bad at the community level. For example, all farmers are instructed to release shrimp fry and to prepare their ponds at the same time in order to control disease outbreaks. However, farmers personally decide to release shrimp fry before or after the instructed cropping time. They do this because if all people raise shrimp at the same time, the prices of shrimp fry will be increased while shrimp products will decrease because of the interplay of supply and demand.

Moreover, in shrimp farming, water resources are a decisive factor for a successful shrimp crop. However, due to the unplanned system of water supply for shrimp ponds, this resource is easily contaminated by the practices of individual households. This implies an internal conflict among farmers regarding the use of water for shrimp farming. In Ca Mau, the conflict is between extensive and intensive shrimp farmers. Although there are regulations for intensive farmers regarding the dredging of their ponds, it is hard to check their compliance. Extensive shrimp farmers often claim that because of intensive farmers’ lack of compliance, waste water leaks out from their intensive ponds. Likewise, in Long An Province, because people practice advanced shrimp culture they have to invest a lot in shrimp food. Although there are regulations on draining water, in their pursuit of individual benefits, people do not follow the regulations. For example, when someone’s shrimp die out because of disease, according to regulations, that individual has to use chemicals to sterilize the pond before draining water out of the pond. However, people often do not do this due to high costs associated with purchasing the relevant chemicals. If neighboring farmers use this infected water, their shrimp would likely catch the disease. As another example, the waste water from a farmer’s shrimp pond can damage his/her neighbors’ ponds, especially of those which are farthest from canals. In both communities, the layout of shrimp ponds depends on individual households. Therefore, there are no separate channels for fresh water inflow and waste water outflow. People get water from and release it into the same canals. This condition is conducive to the outbreak of shrimp epidemics.

## Conclusion

In the shrimp industry, it is obvious that shrimp farmers are vulnerable in their market production. Although various policies exist which aim to promote shrimp production, there is no policy relating to the output of shrimp products. Farmers have to rely on the network of middlemen who always try to make use of shrimp farmers in the whole process of production. With the shift from rice to shrimp, the Mekong Delta farmers are obviously risk-takers who readily engaged in market production. However, working in uncertain conditions, shrimp farmers are not rational actors who always take risks regardless of their conditions. Instead, in their production, shrimp farmers always try to mitigate or disperse their risks. For example, farmers do not strictly observe the local governments’ technical instructions of shrimp farming, which require they invest much capital on shrimp production but do not prove to bring a remarkable success. This is why shrimp production is always judged with such terms as “spontaneous”, “unprofessional” and “small-scale”.

This paper attempts to present the social and ecological challenges of shrimp farming in Vietnam, which is becoming more and more market-oriented. Through the case of shrimp farming, it is obvious that market production could bring immediate benefits for farmers; however, it is also associated with new ecological and social tensions, which people cope with by relying on various strategies. Risk mitigation in shrimp production and labor migration are the two important strategies of local inhabitants for securing their livelihoods. Shrimp farming is a social process in which humans constantly shape and interact with their environment. Water pollution and poor quality post-larvae shrimp are direct consequences of market-oriented production. These results, in turn, lead to negative impacts on shrimp production. Moreover, in the context of poor management and weak enforcement mechanisms, although de facto land ownership is a favorable condition for market oriented shrimp farming, because of individualistic rationality, household production proves to be inappropriate for sustainable production.

### Research methods and case study sites

This paper is based on ethnographic fieldwork undertaken in 2009–2010 in two shrimp raising communities of the Mekong Delta; one in the lower part (Hoa My commune, Cai Nuoc district, Ca Mau Province) and the other in the upper part of the Delta (Tan Chanh commune, Can Duoc district, Long An province). These two communities engage in extensive and advanced extensive shrimp culture, two major types of shrimp culture in the Mekong Delta. Extensive shrimp culture (colloquially called traditional extensive culture) is characterized by low capital investment in pond building, pond preparation, and shrimp raising techniques. Shrimps are left to grow naturally, without being fed processed food. Shrimp fry (post larvae) are released at low density, from one to two shrimps/ m^2^. Shrimps rely completely on natural food. In the whole raising process, farmers only apply such simple pond-preparation measures as using *derris elliptica* to kill the various fish competing with shrimps for food or killing shrimp fry, using lime powder (CaCO3) to reduce alum, and occasionally using chemical fertilizers such as NPK to create algae as a natural food for shrimps in the pond. Having a large amount of land but limited capital, farmers apply these measures only to some extent, not as intensively as suggested by the government’s aquaculture experts. In contrast to other types of shrimp culture in which shrimp fry are released and harvested once per crop, in the extensive type, people usually release fry every one or two months and harvest shrimps every day or every one or two weeks.

With a much improved technique over extensive shrimp culture, the advanced extensive type requires considerable investment in both capital and raising methods. Besides applying more intensively the measures as in extensive culture, farmers adopting the advanced extensive type also use processed pellets or self-processed food and vitamins to feed shrimps, as well as various chemicals such as sterilizer and bio-enzymes in pond preparation and shrimp raising. Pond dikes and ditches are prepared annually. In principle, the density of shrimp post larvae is from three to five shrimps/ m^2^. However, in Long An, in order to have high productivity despite limited land, people release fry with a stock density much higher than the encouraged level, from 10 to 20 shrimps/ m^2^. Besides the two aforementioned types of shrimp culture, a minority of famers also practice industrial shrimp farming which requires a much higher capital investment, normally 40 times higher than the extensive type and five times higher than the advanced extensive type per ha per year.

My research applies quantitative and qualitative methods to collect data. Quantitative methods are employed to generate general patterns and qualitative methods are used to get more detailed data, explanations for quantitative methods. Quantitative data from 354 households provide background information of socio-economic conditions and general picture of shrimp production in both study sites. Qualitative data from 72 interviews and participant observations reveal in-depth information on farmers’ dynamics, analyses, and evaluation.

I apply random sampling method with the margin of sampling errors of 5% or less (Bernard 1994, p.79) and random probability sampling method to select my survey samples. Accordingly, I choose 202 out of 409 households in Thi Tuong hamlet of Ca Mau province and 152 out of 245 households in Dinh hamlet of Long An province. On the basis of sex, age, economic background variables, I select household members, community elites, local authorities and other stakeholders in shrimp farming for in-depth interviews. Totally, we conduct 72 interviews and informal conversations relating to such topics as the shift from rice to shrimp, policies toward shrimp farming, market network, ecological and social changes with farmers who hold various positions in the community. Observations are made at shrimp production activities, rituals and meetings of study communities in order to check, to understand more and to have hints for further interviews.

In addition to the ethnographic fieldwork, this paper is also based on observations of shrimp farming in five other provinces, namely Ben Tre, Tra Vinh, Soc Trang, Bac Lieu, Kien Giang of the Mekong Delta since 2006.

## Authors’ information

Dr. Ngo Thi Phuong Lan is an anthropologist at the University of Social Sciences and Humanities, Vietnam National University – Ho Chi Minh City. Her main research areas are livelihoods, cultural ecology, economic anthropology and agriculture.
